# Supplementary material for: The 40 kDa Linear Polyethylenimine Inhibits Porcine Reproductive and Respiratory Syndrome Virus Infection by Blocking Its Attachment to Permissive Cells
Source: Viruses. 2019 Sep 19;11(9):876. doi: 10.3390/v11090876 (PMC6784015; doi:10.3390/v11090876)
Supplement: Supplementary file 1 [file viruses-11-00876-s001.pdf]

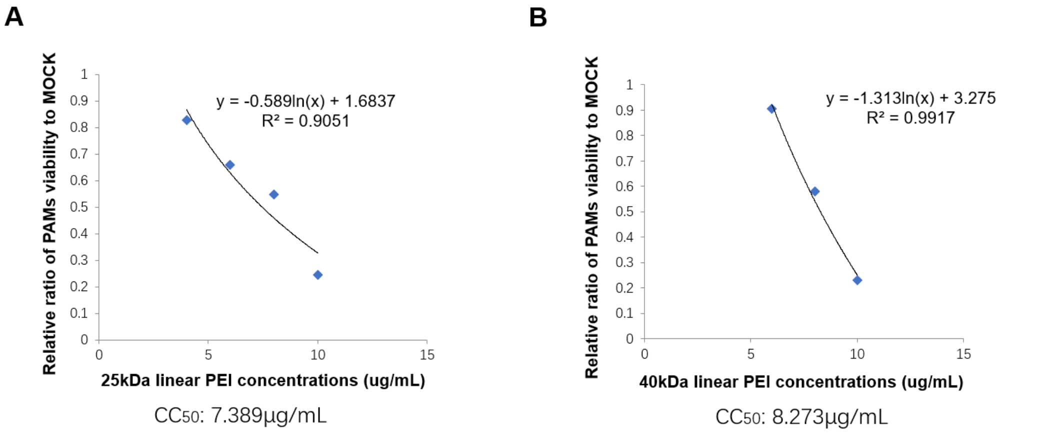

Figure S1. Calculation of cytotoxicity concentration 50% (CC_{50}) for two linear Polyethylenimines (PEIs) in PAMs. (A). Calculation of CC_{50} for 25 kDa linear PEI based on the cytotoxicity assay on PAMs. (B). Calculation of CC_{50} for 40 kDa linear PEI based on the cytotoxicity assay on PAMs.

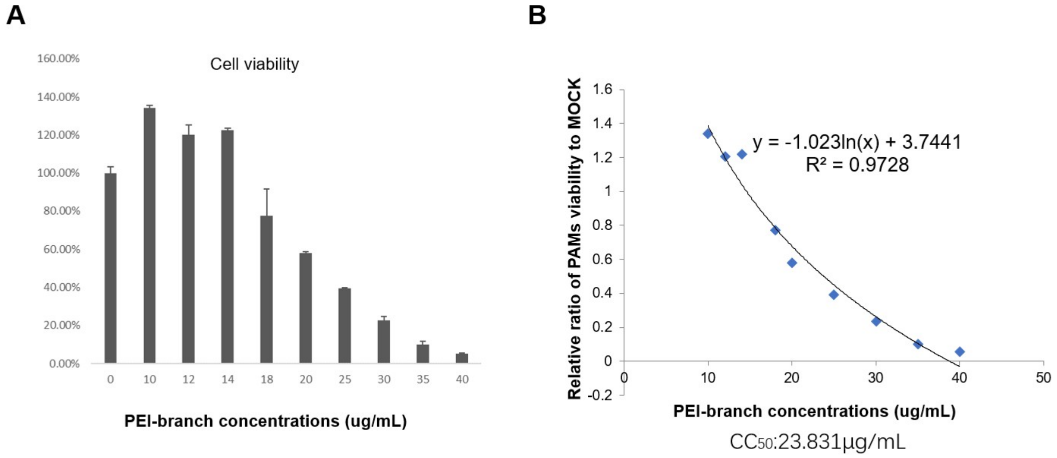

Figure S2. Evaluation of cytotoxicity of branch PEI in PAMs and calculation of CC_{50}. (A). Cell viability assay of PAMs cells incubated in the presence of PEI-branch at the indicated concentrations at 37°C for 24 h. (B) Calculation of CC_{50} for PEI-branch based on the cytotoxicity assay on PAMs

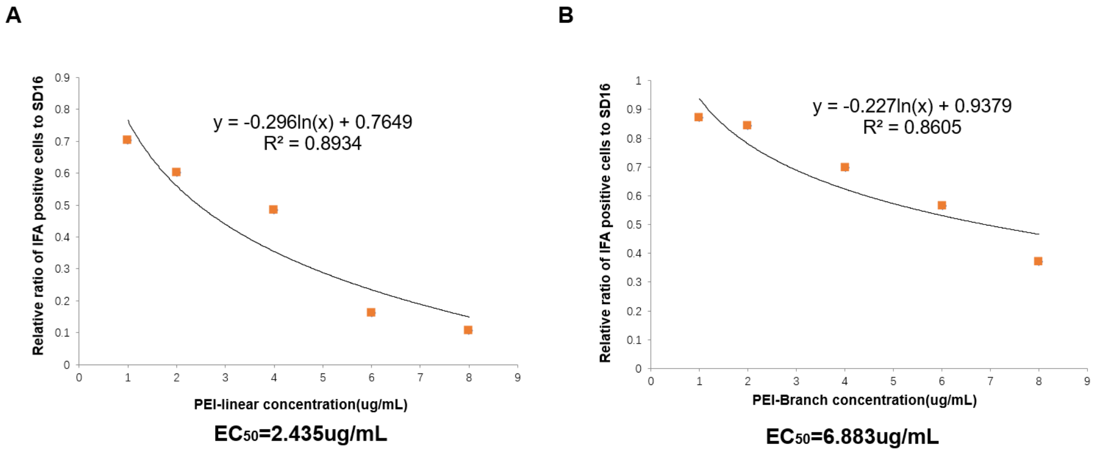

Figure S3. Calculation of median effective concentration (EC_{50}) of PEI in MARC-145 cells. (A). Calculation of EC_{50} for inhibition of PRRSV replication in MARC-145 cells by 40 kDa linear PEI based on the quantification of IFA-positive cell from IFA image via ImageJ software. (B) Calculation of EC_{50} for inhibition of PRRSV replication in MARC-145 cells by branch PEI based on the quantification of IFA-positive cells from IFA images via ImageJ software.
